# Cumulative Association of Obstructive Sleep Apnea Severity and Short Sleep Duration with the Risk for Hypertension

**DOI:** 10.1371/journal.pone.0115666

**Published:** 2014-12-22

**Authors:** Pascaline Priou, Marc Le Vaillant, Nicole Meslier, Audrey Paris, Thierry Pigeanne, Xuan-Lan Nguyen, Claire Alizon, Acya Bizieux-Thaminy, Laurene Leclair-Visonneau, Marie-Pierre Humeau, Frédéric Gagnadoux, for the IRSR sleep cohort group

**Affiliations:** 1 Université d'Angers, Département de Pneumologie, CHU, Angers, France; 2 INSERM 1063, Angers, France; 3 CERMES, CNRS UMR8211 - Inserm U988 - EHESS, Villejuif, France; 4 Service de Pneumologie, Centre Hospitalier, Le Mans, France; 5 Unité de Pneumologie, Pôle santé des Olonnes, Olonne sur Mer, France; 6 Centre d'Etude et de Traitement des Troubles du Sommeil de Saint-Antoine (CETTSA), Hôpital Saint-Antoine, Groupe Hospitalier de l'Est Parisien, Paris, France; 7 Service de Pneumologie, Centre Hospitalier, Cholet, France; 8 Service de Pneumologie, Centre Hospitalier, La Roche sur Yon, France; 9 Service d'Explorations Fonctionnelles, Hôpital Laennec, Nantes, France; 10 Pneumologie, Nouvelles Cliniques Nantaises, Nantes, France; Osaka University, Japan

## Abstract

Obstructive sleep apnea (OSA) and short sleep duration are individually associated with an increased risk for hypertension (HTN). The aim of this multicenter cross-sectional study was to test the hypothesis of a cumulative association of OSA severity and short sleep duration with the risk for prevalent HTN. Among 1,499 patients undergoing polysomnography for suspected OSA, 410 (27.3%) previously diagnosed as hypertensive and taking antihypertensive medication were considered as having HTN. Patients with total sleep time (TST) <6 h were considered to be short sleepers. Logistic regression procedures were performed to determine the independent association of HTN with OSA and sleep duration. Considering normal sleepers (TST ≥6 h) without OSA as the reference group, the odds ratio (OR) (95% confidence intervals) for having HTN was 2.51 (1.35–4.68) in normal sleepers with OSA and 4.37 (2.18–8.78) in short sleepers with OSA after adjustment for age, gender, obesity, diabetes, depression, current smoking, use of thyroid hormones, daytime sleepiness, poor sleep complaint, time in bed, sleep architecture and fragmentation, and study site. The risk for HTN appeared to present a cumulative association with OSA severity and short sleep duration (p<0.0001 for linear trend). The higher risk for HTN was observed in short sleepers with severe OSA (AHI ≥30) (OR, 4.29 [2.03–9.07]). In patients investigated for suspected OSA, sleep-disordered breathing severity and short sleep duration have a cumulative association with the risk for prevalent HTN. Further studies are required to determine whether interventions to optimize sleep may contribute to lower BP in patients with OSA.

## Introduction

Systemic hypertension (HTN) is a highly prevalent condition associated with significant morbidity, increased mortality and high economic cost. Clinic blood pressure (BP) presents an independent continuous relationship with the incidence of stroke, myocardial infarction, sudden death, heart failure and peripheral artery disease as well as end-stage renal disease [1]. There has been growing evidence in support of an independent association between sleep disorders and HTN.

Obstructive sleep apnea (OSA) is strongly associated with HTN. A dose-response relationship between OSA severity and HTN has been well documented by cross-sectional and longitudinal studies in both community- and clinic-based populations [2]–[6]. The most recent meta-analysis based on 28 randomized, controlled trials including 1,948 patients with OSA demonstrated a modest but significant reduction in systolic (≈ −2.6 mmHg) and diastolic (≈ −2.0 mmHg) BP under continuous positive airway pressure (CPAP) therapy [7]. There is growing evidence that the diagnosis of an association between OSA and HTN, as well as the need for their combined treatment, should be considered, particularly in patients with refractory HTN [8]–[11]. Sympathetic overactivity is considered to be a key factor in the pathogenesis of OSA-associated HTN [12], [13].

Sleep habits are influenced by a variety of social, behavioral and environmental factors. The proportion of short sleepers declaring 6h or less of sleep has approximately doubled from about 15 to 30% in the USA over the past four decades [14]. In healthy volunteers, 5 nights of partial sleep deprivation (<5 h) are sufficient to cause a significant increase in sympathetic activity and endothelial dysfunction [15]. Many epidemiological studies have examined the link between sleep duration and HTN in the general population and have provided evidence in support of an independent association between short sleep duration and a higher risk for prevalent and incident HTN (see [16] for systematic review and meta-analysis).

Both OSA and short sleep duration are therefore individually associated with a mild to moderate increase in the risk for HTN. Whether the combination of short sleep duration and OSA confers a stronger association with increased risk for HTN remains to be evaluated. The aim of this multicenter cross-sectional study was to test the hypothesis of a cumulative association of sleep-disordered breathing (SDB) severity and short sleep duration with the risk for prevalent HTN in a large sample of patients investigated for clinical suspicion of OSA.

## Methods

### Ethics statement

This multicenter cross-sectional study was conducted on the *Institut de Recherche en Santé Respiratoire des Pays de la Loire [IRSR] sleep cohort*. Approval was obtained from the University of Angers ethics committee and the "*Comité Consultative sur le Traitement de l'Information en matière de Recherche dans le domaine de la Santé* [C.C.T.I.R.S.] (07.207 bis). The database is anonymous and complies with the restrictive requirements of the "*Commission Nationale Informatique et Liberté* [C.N.I.L.], the French information technology, and personal data protection authority. All patients included in the *IRSR sleep cohort* have given their written informed consent.

### Design and study population

Since May 15, 2007, consecutive patients ≥18 years investigated by overnight polysomnography (PSG) or overnight respiratory recording for suspected OSA in 7 centers from the west of France are eligible for inclusion in the *IRSR sleep cohort*. Patients with mental retardation, who are unable to fill in the questionnaires, or read and/or speak French, and patients with neuromuscular diseases are excluded from the *IRSR sleep cohort*. From the original *IRSR sleep cohort* (n = 7,602), 2,648 patients were investigated by overnight PSG between May 15, 2007 and September 30, 2013. Data on BP measurement and antihypertensive treatment were available for 2,270 patients of whom 1499 had all polysmonographic data available on sleep latency, continuity and architecture, and were included in the present study.

### Measurements, questionnaires and sleep studies

Each patient enrolled in the *IRSR sleep cohort* completed surveys including anthropometric data, smoking habits, alcohol consumption, medical history, and medication use, and underwent fasting blood glucose and glycated hemoglobin assays. Clinic BP was measured in the evening, ≈2 hours before the start of the sleep recording using a periodically calibrated mercury sphygmomanometer. The recorded BP was the average of 3 consecutive readings during a 5-min period following at least 5 min of rest in the sitting position. Obesity was defined by a body mass index (BMI) ≥30 kg/m2. Patients with fasting blood glucose >126 mg/dL or glycated haemoglobin ≥6.5% and/or receiving antidiabetic treatment were considered to present diabetes. Excessive daytime sleepiness (EDS) was defined by an Epworth Sleepiness Scale (ESS) ≥11 [17]. Depression was defined by a QD2A score ≥7 [18] and/or the use of antidepressant medication. Patients reporting frequent difficulty falling asleep and/or difficulty staying asleep and/or early final awakening were considered to be poor sleepers.

Overnight PSG was performed as previously described [18]. Sleep data were scored manually according to Rechtschaffen and Kales criteria [19]. Patients with total sleep time (TST) <6 h were considered to be short sleepers. Patients with TST ≥6 h were considered to be normal sleepers. This cut-off point has been demonstrated to be independently associated with prevalent and incident HTN in previous population-based studies [20], [21]. SDB were scored manually using recommended criteria at the time of the study [22]. Apnea was defined as a cessation of airflow for ≥10 s. Hypopnea was defined as either *(1)* a≥50% reduction in airflow lasting ≥10 s without a requirement for associated oxygen desaturation or arousal or *(2)* any appreciable reduction in airflow for ≥10 s associated with either a ≥3% decrease in SaO2 or an arousal. OSA was defined by an apnea-hypopnea index (AHI) ≥5. The following commonly used cut-offs for AHI were used to define categories of OSA severity: 5 to <30 (mild to moderate OSA), and ≥30 (severe OSA).

### Primary outcome variable

The primary dependent variable of interest was the presence of HTN. BP measurements taken in the sleep clinic were not used to diagnose HTN, as clinical BP measured on only one occasion cannot be used as a reliable indicator for the definition of HTN [1]. Only patients who were previously diagnosed as hypertensive and were taking antihypertensive medication were considered as having HTN.

### Statistical analysis

All statistical analyses were performed with SAS software (SAS/STAT Package 2002–2003 by SAS Institute Inc., Cary, NC, USA). Patients with and without HTN were compared using Chi-square test for categorical variables and 2-sample t-test for continuous variables. Variables with p value <0.05 were then entered in a logistic regression procedure to determine the independent association of HTN with OSA and sleep duration. In a first step, we separately studied the association of HTN with OSA alone and with short sleep duration alone. In a second step, adjusted odds ratio (OR) (95% confidence intervals [CI]) for HTN associated with short sleep duration were determined in the whole OSA group and then in the 2 categories of OSA severity. Then, we calculated the adjusted OR (95%CI) for HTN associated with various combinations of OSA severity and sleep duration, considering normal sleepers without OSA as the reference group. Results are expressed as percentages, mean (standard deviation) (SD) and OR (95%CI). A 2-tailed p value <0.05 was considered significant.

## Results

Demographics, anthropomorphic, clinical and polysomnographic characteristics of the study population are presented in Table 1. The overall prevalence of HTN was 27.3%. Short sleep duration was observed in 17.2% of patients and OSA was diagnosed in 84.6% of patients including 57.2% of mild to moderate OSA and 42.8% of severe OSA. On univariate analysis, HTN was significantly associated with both OSA severity and short sleep duration. Significant associations were also observed between HTN and age, obesity, diabetes, depression, smoking habits, EDS, use of thyroid hormones, time in bed, sleep latency, wake time after sleep onset (WASO), time in slow wave sleep and the overall arousal index. There was also a trend toward a higher rate of poor sleep complaints in patients with HTN as compared with normotensive subjects (30.8 *vs* 26.0%, p = 0.070). In contrast, no difference was observed between patients with and without HTN for gender, alcohol consumption, use of inhaled beta2-agonists, and time spent in rapid eye movement sleep. As expected, short sleepers had higher sleep latency (p<0.0001), WASO (p<0.0001) and arousal index (p = 0.0013), lower time in bed (p<0.0001) and lower time in slow wave sleep (p<0.0001) as compared with normal sleepers (data not shown). In contrast, the time in rapid eye movement sleep did not differ between short and normal sleepers (p = 0.1884).

**Table 1:** Characteristics of the study population.

Variables	All	HTN	No HTN	P value (HTN *vs* no HTN)
N	1499	410	1089	
Age, years	54.3(13.5)	62.6(10.9)	51.1(13.1)	<0.0001
Female, %	36.3	36.6	36.2	0.8843
BMI, kg/m^2^	23.8(5.9)	31.0(5.8)	28.1(5.8)	<0.0001
Obesity (BMI ≥30 kg/m^2^), %	37.1	53.2	31.1	<0.0001
Diabetes, %	13.5	30.2	7.2	<0.0001
Depression, %	27.6	31.5	26.1	0.0375
Current smokers, %	37.7	26.3	41.0	0.0006
Inhaled beta2-agonists, %	26.4	25.8	26.6	0.8392
Use of thyroid hormones, %	10.4	13.1	9.4	0.0396
Daily alcohol consumers, %	41.3	43.4	40.4	0.3117
Epworth sleepiness scale	10.4(5.1)	9.4(4.9)	10.7(5.1)	<0.0001
EDS, %	48.7	39.3	52.2	<0.0001
Poor sleepers, %	27.3	30.8	26.0	0.0700
Apnea-hypopnea index, n	26.1(22.6)	32.9(23.4)	23.6(21.8)	<0.0001
Sleep parameters				
Time in bed, min	532.9(46.7)	527.4(48.3)	535.0(46.0)	0.0047
Sleep latency, min	23.4(25.8)	27.6(26.8)	21.8(25.3)	<0.0001
WASO, min	85.7(58.8)	104.0(62.8)	78.8(55.7)	<0.0001
Total sleep time, min	423.8(76.5)	395.7(79.9)	434.3(72.4)	<0.0001
Short sleepers, %	17.2	30.1	12.3	<0.0001
REM sleep, % TST	22.1(8.8)	22.4(9.5)	22.0(8.5)	0.5004
SW sleep (N3), % TST	19.5(6.4)	18.9(6.8)	19.8(6.3)	0.0179
Arousal index, n	29.4(16.4)	30.8(16.9)	28.9(16.2	0.0456

Data are expressed as mean (SD) or percentages.

HTN, hypertension; BMI, body mass index; EDS, excessive daytime sleepiness; WASO, wake time after sleep onset; REM, rapid eye movement; SW, slow wave.

Logistic regression models were used to determine the independent association of HTN with OSA severity alone and with sleep duration alone. As shown in Table 2, OSA and short sleep duration were both individually associated with a significant increased risk for HTN after adjustment for age, gender, obesity, diabetes, depression, current smoking, use of thyroid hormones, excessive daytime sleepiness, poor sleep complaint, time spent in slow wave sleep, overall arousal index, time in bed, and study site (model 1). Furthermore, a positive and significant linear trend was observed for the odds of HTN with increasing OSA severity (p = 0.0012). The association of HTN with SDB or sleep duration was only slightly modified when time in bed was successively replaced by sleep latency (model 2) and WASO (model 3). All subsequent multivariable analyses were performed using model 1.

**Table 2:** Multivariate adjusted odds ratio (OR) (95% confidence interval [CI]) for hypertension associated with sleep-disordered breathing or sleep duration.

Variables	Model 1	Model 2	Model 3
	OR (95%CI)	OR (95%CI)	OR (95%CI)
Sleep-disordered breathing			
No OSA	1.00	1.00	1.00
All OSA	2.25 (1.33–3.82)	2.22 (1.31–3.76)	2.27 (1.34–3.85)
Mild to moderate OSA	2.05 (1.21–3.47)	2.01 (1.19–3.39)	2.06 (1.22–3.49)
Severe OSA	2.33 (1.33–4.10)	2.31 (1.32–4.06)	2.35 (1.34–4.12)
P for linear trend†	0.0011	0.0012	0.0010
Sleep duration			
Normal sleepers	1.00	1.00	1.00
Short sleepers	1.72 (1.22–2.44)	1.71 (1.20–2.24)	1.94 (1.31–2.89)

OSA, obstructive sleep apnea.

Model 1 included OSA severity or sleep duration adjusted for age, gender, obesity, diabetes, depression, current smoking, use of thyroid hormones, excessive daytime sleepiness, poor sleep, time spent in slow wave sleep, overall arousal index, time in bed, and study site.

Model 2 included OSA severity or sleep duration adjusted for age, gender, obesity, diabetes, depression, current smoking, use of thyroid hormones, excessive daytime sleepiness, poor sleep, time spent in slow wave sleep, overall arousal index, sleep latency, and study site.

Model 3 included OSA severity or sleep duration adjusted for age, gender, obesity, diabetes, depression, current smoking, use of thyroid hormones, excessive daytime sleepiness, poor sleep, time spent in slow wave sleep, overall arousal index, wake time after sleep onset, and study site.

Adjusted OR were all statistically significant with p value <0.01

† Tested by the Cochrane-Armitage trend test.

As shown in Table 3, OSA patients with short sleep duration had a significant increased risk for HTN after adjusting for confounders as compared with OSA patients with normal sleep duration (p = 0.0056). The increased risk for HTN associated with short sleep duration was also significant in patients with mild to moderate OSA (p = 0.0459) and in patients with severe OSA (p = 0.0256)

**Table 3:** Multivariate adjusted Odds Ratio (OR) (95% confidence interval [CI]) for hypertension associated with sleep duration in the whole obstructive sleep apnea (OSA) group and in the 2 categories of OSA severity.

Sleep duration	Normal sleepers	Short sleepers
SDB		
All OSA (n = 1269)	1.0	1.66 (1.16–2.38)**
Mild to moderate OSA (n = 725)	1.0	1.68 (1.01–2.81)*
Severe OSA (n = 544)	1.0	1.84 (1.08–3.15)*

SDB, sleep-disordered breathing.

OR were adjusted for age, gender, obesity, diabetes, depression, current smoking, use of thyroid hormones, excessive daytime sleepiness, poor sleep, time spent in slow wave sleep, overall arousal index, time in bed, and study site.

* p<0.05.

**p<0.01.

**Figure 1:**
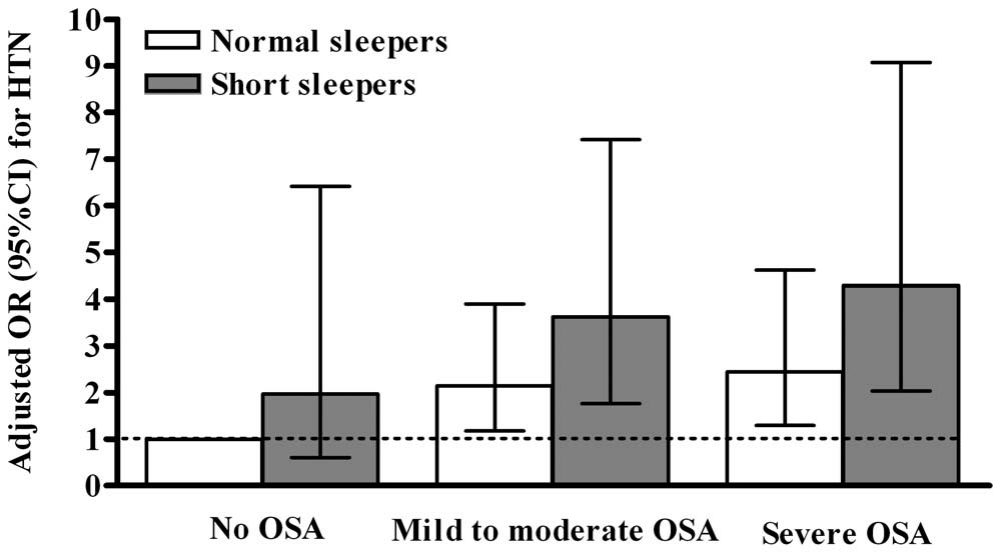
Multivariate adjusted odds ratio (OR) (95% confidence interval [CI]) for hypertension (HTN) associated with various combinations of sleep-disordered breathing and sleep duration. OSA, obstructive sleep apnea. OR were adjusted for age, gender, obesity, diabetes, depression, current smoking, use of thyroid hormones, excessive daytime sleepiness, poor sleep, time spent in slow wave sleep, overall arousal index, time in bed, and study site. P<0.0001 for linear trend according to the Cochrane-Armitage trend test.

Considering normal sleepers without OSA as the reference group the adjusted OR (95%CI) for having HTN was 2.51 (1.35–4.68) in normal sleepers with OSA and 4.37 (2.18–8.78) in patients with OSA and short sleep duration. Figure 1 presents the adjusted OR for HTN associated with 6 combinations of OSA severity and sleep duration. The risk for HTN appeared to present a cumulative association with OSA severity and short sleep duration (p<0.0001 for linear trend). The higher risk for HTN was observed in patients with both severe OSA and short sleep duration (OR, 4.29 [2.03–9.07]).

## Discussion

This large cross-sectional study in 1,499 patients investigated by PSG for suspected SDB shows a cumulative association of OSA severity and short sleep duration with the risk for HTN. After adjustment for confounders, the risk for HTN was synergistically increased among patients with both short sleep duration and mild to moderate or severe OSA. Severe OSA with short sleep duration was associated with a more than 4-fold increased risk for HTN as compared with normal sleepers without OSA. Our findings provide evidence that, in addition to the AHI, objectively measured sleep duration could be clinically relevant for assessing vascular involvement in patients with OSA.

Experimental studies of sleep deprivation have demonstrated significant increases in BP and sympathetic nervous system activity after nights with restricted sleep duration both in normotensive [15], [23] and hypertensive subjects [24]. A recent meta-analysis was conducted to clarify the association between sleep duration and HTN risk in real life conditions [16]. The pooled results indicated that short sleep duration was associated with a 21% increase in the risk of prevalent HTN, and a 23% increase in the risk of incident HTN. In most studies, sleep duration was self-reported by questionnaires. A significant association was also observed between HTN and long sleep duration (≥9 h) in cross-sectional but not in longitudinal studies [16].

Few studies have evaluated the association between objective sleep data as measured by sleep recordings and HTN. In 1,741 adults from the Penn State Cohort, insomnia with PSG-measured short sleep duration (<6 h) was independently associated with both prevalent [20] (OR: 3.5, 95% CI: 1.6–7.9) and incident [21] HTN (OR: 3.8, 95% CI: 1.6–9.0). The association remained significant after adjustment for SDB, but only 10% of the population had OSA as defined by an AHI ≥5. In 784 community-dwelling, ambulatory men ≥65 years, incident HTN was inversely associated with the percentage of slow wave sleep after adjustment for sleep duration, sleep fragmentation and SDB, but the mean respiratory disturbance index was only 10 [25].

Little is known about the association of sleep duration with the risk for HTN in clinical populations of OSA. In a retrospective study of 312 patients investigated by PSG, 150 of whom received a diagnosis of OSA, Ucar et al. found lower sleep duration in patients with HTN than in subjects with no comorbidity [26]. When the analysis was restricted to OSA patients, sleep duration ≤6 h, as assessed by a telephone-administered questionnaire, was significantly associated with coronary heart disease, but not with HTN. A recent case-control study demonstrated that OSA patients with resistant HTN (n = 62) had shorter sleep duration than subjects with either controlled HTN (n = 49) or normotension (n = 40) after controlling for SDB severity, and adjusting for differences in demographics, anthropometric, and medical factors among the groups [27]. To the best of our knowledge, the present study is the first to demonstrate a cumulative association of OSA severity and short sleep duration with HTN in a large population of patients investigated for clinical suspicion of OSA. The present study was not designed to determine the factors leading to shorter sleep duration in some of our OSA patients. These factors are likely to be multiple as sleep habits are influenced by a variety of social, behavioral and environmental factors. As previously suggested, short sleep duration in OSA patients may represent a consequence of underlying poor health and may also represent a brain defence mechanism [28]. Nevertheless, our study provides evidence that regardless of SDB severity, OSA patients with short sleep duration are at higher risk for prevalent HTN as compared with OSA patients with normal sleep duration. Further studies are required to investigate the underlying mechanisms linking the combination of OSA and short sleep duration with HTN. However, it can be assumed that short sleep duration [15], [23], [24] and OSA-associated intermittent hypoxia [13] and sleep fragmentation [29] exert synergistic effects on sympathetic tone leading to sustained daytime sympathetic overactivity and elevated BP. Interestingly, we found that patients with HTN were less likely to be sleepy than normotensive subjects although SDB was more severe in the HTN group. A recent study in OSA patients with heart failure demonstrated an inverse relationship between subjective daytime sleepiness and sympathetic activity, suggesting that daytime sympathetic overactivity may counteract OSA-induced EDS [30]. It can be hypothesized that hypertensive patients from our cohort were less sleepy due to higher sympathetic activity.

As recommended by clinical guidelines [31], respiratory recordings during sleep are widely used in patients with high pretest probability of moderate to severe OSA. Our findings that the risk for HTN was synergistically increased in patients with both OSA and short sleep duration suggest that objective sleep measurements may provide clinically relevant information to assess the medical severity of OSA, even in patients with moderate or severe SDB. Systematic reviews and meta-analyses of randomized controlled trials showed that older age, poorer CPAP adherence and lack of EDS at OSA diagnosis are associated with lower BP reduction on CPAP [7]. Among men, sleep time decreases an average of 27 min per decade from midlife until the eighth decade [32]. It can be assumed that short sleep duration may contribute to higher sympathetic activity, lower treatment use, and lower BP reduction on CPAP.

Some limitations should be taken into account when interpreting our findings. The cross-sectional design of the study does not allow any conclusions to be drawn regarding the causal pathway of these associations. However, previous experimental studies of intermittent hypoxia and sleep deprivation provide strong arguments in support of a causal link [13], [15]. It should be also noted that most cross-sectional studies that established the association of sleep disorders with HTN [3], [20] were later confirmed by data from prospective studies [4], [21]. We acknowledge that our study was performed on a clinic-based sample. However, our sample of patients can be assumed to describe a “typical” pattern of OSA patients, as the present study included a significant number of subjects with a wide range of disease severity. Future studies should be completed in general population samples to confirm this finding. Although BP variability is of clinical importance in OSA, we acknowledge that we did not perform ambulatory BP monitoring (ABPM) in the present study. Our multicenter cohort study was based on routine clinical practice for the management of OSA patients. Clinic BP measurement remains the ‘gold standard’ for screening, diagnosis and management of HTN [1] and routine use of ABPM in OSA patients is not currently recommended by French Practice Guidelines [33]. As clinical BP was measured on only one occasion in our patients we did not use this single measurement for the definition of HTN. Only patients who were previously diagnosed as hypertensive and were taking antihypertensive medication were considered as having HTN. In a recent historical cohort study including 10,149 patients who underwent PSG for suspect OSA [34], previously diagnosed HTN was an independent predictor of cardio-vascular events and all-cause mortality. Of note, low sleep duration on baseline PSG was also an independent predictor cardio-vascular events and all-cause mortality in this cohort. Another potential limitation of our study is that sleep duration was measured during a single night PSG, which may not be representative of the subject's habitual sleep pattern. Actigraphy could constitute a simpler alternative tool to objectively measure sleep duration for a period of days. A good level of agreement has been observed between actigraphy and PSG for sleep duration measurement in OSA [35]. A previous study using home actigraphy for 3 consecutive days found that reduced sleep duration was predictive of higher BP levels [36].

## Conclusion

In patients with clinical suspicion of OSA, sleep-disordered breathing severity and short sleep duration have a cumulative association with the risk for prevalent HTN. Further studies are required to determine whether interventions to optimize sleep may contribute to lower BP in patients with OSA.
